# HaplotypeTools: a toolkit for accurately identifying recombination and recombinant genotypes

**DOI:** 10.1186/s12859-021-04473-1

**Published:** 2021-11-22

**Authors:** Rhys A. Farrer

**Affiliations:** Medical Research Council Centre for Medical Mycology at the University of Exeter, Exeter, UK

**Keywords:** Haplotype, Phasing, Recombination, Software, *Batrachochytrium dendrobatidis*, Hybridization, Recombination

## Abstract

**Background:**

Identifying haplotypes is central to sequence analysis in diploid or polyploid genomes. Despite this, there remains a lack of research and tools designed for physical phasing and its downstream analysis.

**Results:**

HaplotypeTools is a new toolset to phase variant sites using VCF and BAM files and to analyse phased VCFs. Phasing is achieved via the identification of reads overlapping ≥ 2 heterozygous positions and then extended by additional reads, a process that can be parallelized across a computer cluster. HaplotypeTools includes various utility scripts for downstream analysis including crossover detection and phylogenetic placement of haplotypes to other lineages or species. HaplotypeTools was assessed for accuracy against WhatsHap using simulated short and long reads, demonstrating higher accuracy, albeit with reduced haplotype length. HaplotypeTools was also tested on real Illumina data to determine the ancestry of hybrid fungal isolate *Batrachochytrium dendrobatidis* (*Bd*) SA-EC3, finding 80% of haplotypes across the genome phylogenetically cluster with parental lineages *Bd*GPL (39%) and *Bd*CAPE (41%), indicating those are the parental lineages. Finally, ~ 99% of phasing was conserved between overlapping phase groups between SA-EC3 and either parental lineage, indicating mitotic gene conversion/parasexuality as the mechanism of recombination for this hybrid isolate. HaplotypeTools is open source and freely available from https://github.com/rhysf/HaplotypeTools under the MIT License.

**Conclusions:**

HaplotypeTools is a powerful resource for analyzing hybrid or recombinant diploid or polyploid genomes and identifying parental ancestry for sub-genomic regions.

## Background

DNA sequence analysis based on alignments to unphased diploid or polyploid genome assemblies can result in errors and misleading results [1]. These errors will scale with the abundance of heterozygosity. Such reference genomes and accompanying gene annotation typically consist of haploid sequences representing a ‘patchwork’ of haplotypes (nucleotides that co-occur in a single chromosome), and thus, any given base may derive from either chromosome. Genome sequencing and alignment rarely distinguishes variants found together (cis) or on homologous chromosomes (trans), and therefore neglects to identify the allelic variation of genes, and instead describes only the sum of all variants in both homologous genes. In the most extreme case of two or three heterozygous positions co-localizing in a codon, nonsense or readthrough mutations may be unidentified or misidentified through lack of or inaccurate phasing information. For example, the codon WGW (where W = IUPAC for Weak bond = A or T) reflects either AGA (Arg) and TGT (Cys) or AGT (Ser) and TGA (Stop) in the standard genetic code.

Identifying haplotypes (nucleotides in phase) is central to determining genotype–phenotype associations from heterozygous alleles, identifying recombinant or hybrid isolates in microbial populations, and determining parental ancestry. Haplotyping is also a precursor to a range of genetic attributes including effective population size, signatures of selection and evolution e.g. Integrated Haplotype Scores and Extended Haplotype Homozygosity tests [2]. Haplotyping can reveal candidate genes for mendelian disorders [3], given haplotype variation impacts gene expression [4], splice variants, folding and function [5]. For example, hundreds of ‘phase-sensitive’ human genes have been identified, including olfactory receptors and proteins related to the immune system such as the MHC (Class I and II) which contain two or more potentially functionally significant mutations that can reside in either cis or trans configurations. Thus, the phase of many mutations is likely to be of critical importance for protein function, phenotype and clinical genome interpretation. Ongoing initiatives to understand the associations of haplotypes with human disease include the HapMap Project [6] and the Genomics England’s 100,000 Genomes Project [7]. Haplotyping in non-model organisms including diverse Emerging Fungal Pathogens remains largely unexplored [8].

Despite the importance of haplotyping, there remains a lack of research into the physiological consequences of having variants co-reside on chromosomes, or distributed across two homologues chromosomes [9]. Research using haplotypes is limited for a variety of reasons including the computational complexity of haplotyping, lack of haplotyping tools, lack of tools to perform analysis of those haplotypes, and increased complexity of haplotype analysis given the extra step of phasing and its imperfect outputs. Current methods that have been developed to phase sequence data include experimental phasing methods, along with computational phasing with related individuals and computational phasing with unrelated individuals [10]. Experimental phasing is achieved by direct sequencing encompassing two or more heterozygous genotypes of an individual, while the other methods rely on a priori knowledge of haplotypes, or modelling haplotypes based on factors such as patterns of linkage disequilibrium [11]. Recombination maps are often not known or challenging to infer particularly in non-model organisms. Experimental phasing is limited by sequence read-length, and is not feasible on genomes lacking sufficiently heterozygous and proximal variants. However, the deep coverage of next-generation sequencing and paired-end information can cheaply and confidently yield haplotypes in a wide range of genomes.

Experimental phasing has been implemented by several tools, including some under active development. For example, experimental phasing is part of the GATK HaplotypeCaller algorithm [12], which forms part of its genotyping algorithm. GATK stores the physical phasing information in Pre-Implantation Genetic Testing (PGT) and Physical phasing ID information (PID) format fields of the VCF [13]. Other tools specifically designed to perform physical phasing for large and accurate haplotype construction include WhatsHap [14], HapCut2 [15], and SmartPhase [16]. The underlying algorithms of each method include weighted minimum error correction, maximum-likelihood, and read-based (either RNAseq or DNAseq) phasing respectively. HapCut2 works on a range of sequencing data including Hi-C and long read sequencing [15]. WhatsHap takes a dynamic programming approach that is both fast and more accurate than statistical phasers [14]. PoolhapX infers haplotypes across naturally pooled samples [17]. Accuracy for these tools has been determined by consensus to other methods or using simulated data. One potential drawback for each of these tools is that there are not easily (no options to) parallelize across multiple nodes on a computer cluster. Where such resources are available, this approach may decrease computational time.

Here, I present a toolset to phase diploid variant calls from whole genome sequencing data, validating phasing accuracy, phylogenetically placing haplotypes to other lineages or species, and identifying crossovers between pairs of phased VCFs. HaplotypeTools phasing performed better overall in terms of accuracy, at the cost of smaller haplotypes, in comparison to WhatsHap [14]. Both tools performed considerably better than GATK HaplotypeCaller physical phasing alone. HaplotypeTools was also used to identify the parental lineages and loci of crossovers for a hybrid fungal isolate belonging to the species *Batrachochytrium dendrobatidis*.

## Results

### Benchmarking with simulated data

HaplotypeTools was benchmarked against WhatsHap using simulated reads from the genome of the fungal pathogen *Batrachochytrium dendrobatidis* (*Bd*) JEL423 (see methods), highlighting several differences between the tools. First, the accuracy of the variant caller Pilon [18] to call heterozygous positions from short (100nt) and long-read (10 kb) paired end alignments (20X depth) was assessed (Table 1) revealing high levels of sensitivity (> 0.91), specificity (> 0.99) and overall accuracy (> 0.98), which is suitable for testing downstream phasing.

**Table 1:** Accuracy of heterozygous variant calling by Pilon was assessed

Test	1	2	3	4	5	6
Introduced HET	23,13,700	23,13,700	2,31,370	2,31,370	23,137	23,137
Introduced HET (/kb)	100	100	10	10	1	1
Read Length (nt, paired)	100	10,000	100	10,000	100	10,000
SNP	86,808	1,89,498	7,752	16,583	1,075	1,633
HET	20,90,044	17,81,654	2,30,380	1,91,558	37,153	22,365
INS	43	66	0	0	0	0
DEL	48	69	2	1	2	1
AMB	2	92	2	59	2	67
TP	20,27,722	17,45,567	2,13,362	1,87,637	21,429	18,807
TN	2,08,11,068	1,97,41,846	2,28,43,007	2,19,30,208	2,30,49,406	2,20,49,041
FP	62,322	36,087	17,018	3,921	15,724	3,558
FN	98,679	1,65,971	9,073	16,227	904	1,572
FP other	86,901	1,89,725	7,756	16,643	1,079	1,701
TP (%)	87.64	75.44	92.22	81.10	92.62	81.29
TN (%)	89.94	85.32	98.73	94.78	99.62	95.29
FP (%)	2.98	2.03	7.39	2.05	42.32	15.91
FN (%)	4.26	7.17	3.92	7.01	3.91	6.79
Sensitivity	0.95	0.91	0.96	0.92	0.96	0.92
Specificity	0.99	0.99	1.00	1.00	1.00	1.00
Accuracy	0.99	0.98	1.00	1.00	1.00	1.00

Paired reads (100nt or 10 kb) were simulated at 20X depth from reference *Bd* JEL423 genome that was duplicated to create an in silico diploid. In silico mutations were then randomly introduced throughout (1/kb, 10/kb or 100/kb). Reads were aligned to the original reference sequence (non-duplicated, non-mutated version), and diploid variants called by Pilon. Counts of variants are shown including single nucleotide polymorphisms (SNP), heterozygous positions (HET), insertions (INS), deletions (DEL) and ambiguous (AMB). Accuracy was assessed according to Comparison of FDR tool [28], that calculated TN = true negatives (correct reference bases), TP = true positives (correct HET), FN = false negatives (incorrect reference bases) and FP = false positives (incorrect HET). FP (other) is a count of all additional (non-heterozygous) incorrect bases including SNPs, INS, DEL and AMB. > 99% of FP (other) were SNPs. TP (%) and FN (%) are precents of Introduced HET, FN (%) is a percent of assembly length, and FP (%) is a percent of HETs called. Sensitivity = TP/(TP + FN), Specificity = TN/(TN + FP + FP (other)), Accuracy = (TN + TP)/(TN + TP + FN + FP + FP (other))

Haplotypes defined by HaplotypeTools and WhatsHap were assessed for haplotype length, coverage, accuracy and computational time (Table 2). HaplotypeTools outperformed WhatsHap in terms of phasing accuracy, while it underperformed in terms of haplotype length, genome coverage of those haplotypes, sensitivity and QAN50 values (an assessment of haplotype length and quality in terms of Switch Errors). For example, the longest haplotype block/pair from HaplotypeTools was ~ 11.7 kb, compared with ~ 874 kb for WhatsHap.

**Table 2:** Assessment of HaplotypeTools and WhatsHap on simulated paired reads (100nt or 10 kb) from 100/kb, 10/kb and 1/kb heterozygosity levels

Tool	Param	Het. (/Kb)	Read Length (nt, paired)	Hap n	Genome coverage (%)	Hap N_max_	Hap N_50_	SE	SER	QAN50	Time
HT	default	100	100	95,532	83.2	2867	342	6248	0.0031	248	27 m41 s
HT	default	10	100	33,962	12.0	620	109	25	0.0002	N.D	32 m33 s
HT	-m 2	10	100	34,423	12.1	620	109	32	0.0003	N.D	30 m14 s
HT	default	1	100	728	0.2	211	66	0	0.0000	N.D	32 m59 s
HT	-m 2	1	100	875	0.2	211	64	1	0.0005	N.D	29 m42 s
HT	default	100	10,000	61,536	73.3	6455	528	4242	0.0024	267	31 m13 s
HT	-m 2	100	10,000	56,825	80.5	6455	605	4185	0.0022	353	27 m53 s
HT	-m 2 -r 100 kb	100	10,000	56,903	80.3	11,694	612	4185	0.0022	350	23 m13 s
HT	default	10	10,000	5,965	78.6	9995	6480	35	0.0002	4254	29 m14 s
HT	default	1	10,000	2,492	57.7	9946	7774	0	0.0000	5888	27 m
WH	default	100	100	366	95.1	8,43,884	3,08,400	35,461	0.0163	9,91,296	95 m56 s
WH	default	10	100	2,496	92.7	74,839	17,864	1570	0.0070	19,555	14 m29 s
WH	default	1	100	6,540	12.1	3932	578	235	0.0192	N.D	43 s
WH	default	100	10,000	128	89.1	8,43,900	3,60,273	29,373	0.0144	9,36,966	23 m27 s
WH	default	10	10,000	130	90.0	8,74,030	4,00,757	347	0.0016	4,64,693	3 m11 s
WH	default	1	10,000	196	88.0	7,52,451	2,03,122	18	0.0009	97,221	24 s

Haplotype (Hap) count (n) and their coverage across the genome assembly is shown as a percent. Haplotype lengths are described by Hap N_max_ (the maximum haplotype length found) and Hap N_50_ (the shortest haplotype length that includes ≥ 50% of haplotype sequence.). Switch Errors (SE), Switch Error Rate (SER), Quality Adjusted N50 (QAN50) and Computational Time were calculated and compared as described in methods (code to calculate these values are part of HaplotypeTools)

HaplotypeTools achieved higher accuracy overall than WhatsHap according to a range of metrics (Table 2). HaplotypeTools resulted in fewer (< 14%) Switch Errors (SE), and lower Switch Error Rate (SER) for every test, which had a value of between 0 and 0.0031 compared with 0.007 and 0.016 for WhatsHap. Indeed, for two of the tests (100nt reads with 1/kb heterozygosity and 10 kb reads with 1/kb heterozygosity), HaplotypeTools did not produce a single switch error (SER = 0), demonstrating the high accuracy achieved by HaplotypeTools even using default settings.

Lowering the minimum haplotype coverage parameter in HaplotypeTools achieved better SE and SER for one of the tests (10 kb reads for 100/kb heterozygosity). For the same test data, increasing the maximum phasing length resulted in longer haplotypes and reduced computational time, at the cost of a slightly decreased genome coverage (Table 2). Therefore, adjusting HaplotypeTools’ parameters may achieve better results than the default settings depending on the use case (e.g. read length and heterozygosity level) and desired outcome (sensitivity vs specificity).

While optional, HaplotypeTools was designed to run in parallel across a computer cluster – first splitting up the VCF and BAM files into windows that can be processed in parallel. HaplotypeTools was scatter gathered across ~ 100 low-spec nodes (8 Gb RAM, Intel Xeon CPU E5-2680 v2 @ 2.80 GHz), which took between 23m13s and 32m59s till completion (Table 2). WhatsHap is not designed to run in parallel (although such a process could be achieved with a custom pipeline if desired). Thus, WhatsHap was tested locally on a single high-spec laptop (32 Gb RAM, Intel Core i9-9980HK CPU @ 2.40 GHz). WhatsHap required some preprocessing to run (e.g. removing reference bases from VCFs). After pre-processing, WhatsHap was overall computationally faster: taking between 95m56s for 100 nt reads with 100/kb heterozygous positions, to as quickly as just 24 s on 10 kb reads with 1/kb heterozygous positions.

### Results on real data

HaplotypeTools was used to phase real Illumina data (100nt paired reads, 43X depth) to determine the parental lineages of the hybrid *Bd* isolate SA-EC3 with several settings. GATK v4 HaplotypeCaller was used for variant calling, which includes its own physical phasing, and could therefore also be compared to the results from HaplotypeCaller and WhatsHap (Table 3).

**Table 3:** Details of haplotypes from phasing a single-isolate VCF from hybrid *Bd* isolate SA-EC3 using GATK v4 HaplotypeCaller, HaplotypeTools (default settings), and WhatsHap (default settings)

	HaplotypeCaller	HaplotypeTools	WhatsHap
Number of haplotypes	5545	7975	9982
Total phased nucleotides	1,35,334	6,74,408	50,02,469
Haplotype Nmax (nt)	230	1083	8485
Haplotype N50 (nt)	26	115	1107
Haplotype N90 (nt)	13	44	314
Overlap with HT (default) (#haps)	2027	N/A	7948
Overlap with HT (nt)	44,157	N/A	6,58,713
Computational time	N/A	41m56s	5m54s

Rows include the number of haplotypes produced by each tool, the total number of nucleotides included in those haplotypes, the maximum haplotype length found (Haplotype N_max_), the Hap N_50_ and N_90_ (the shortest haplotype length that includes ≥ 50% and ≥ 90% of haplotype sequence, respectively.). The number of haplotypes (#haps) that overlap on the genome assembly with haplotypes produced by HaplotypeTools (HT), and the number of nucleotides (nt) in those haplotpes. Computational time is also given for HaplotypeTools and Whatshap, but omitted for GATK given its primary role was variant calling, which both HaplotypeTools and WhatsHap were also based on, and the time taken for phasing alone can neither be determined or distinguished from that process

**Fig. 1:**
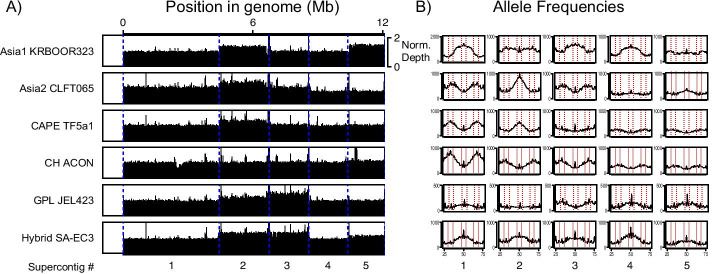
The five isolates representing each of the lineages were assessed for ploidy and aneuploidy (largest five supercontigs presented). **A** Non-overlapping 10 kb windows of the normalized depth of coverage (normalized by total sequencing depth across percentiles of GC content, and excluding ambiguous sites) shows evidence for aneuploidies (supercontig 2, 3 and 5) among each of the *Bd* isolates apart from *Bd*CH ACON. **B** Allele frequencies (percent of reads agreeing with the reference base) are shown from 25% agree to 75% disagree, with red-dotted lines indicating greatest support for bi-allelic/diploidy between 47 and 53% and greatest support for tri-allelic/triploidy between 30–36% and 63–69%

The five isolates representing each of the lineages were assessed for ploidy and aneuploidy. Non-overlapping windows presenting normalized depth of coverage revealed evidence for aneuploidies in all isolates apart from *Bd*CH ACON (Fig. 1). Supercontig (sc) 1 is the largest supercontig and therefore the best evidence for the baseline ploidy from genomic data alone. Therefore, based on this depth of coverage and allele frequencies (percent of reads agreeing with the reference base), Asia1 KRBOOR323 is diploid with sc2 and sc3 trisomies, Asia2 CLFT065 and CAPE TF5a1 are triploid with a sc2 tetrasomy, CH ACON is triploid with no aneuploidies, GPL JEL423 is diploid with a sc3 trisomy, and Hybrid SA-EC3 is diploid with possible sc2 and sc3 tetrasomy. WhatsHap and HaplotypeTools were therefore tested on polyploid and aneuploid genomes, which could impact the accuracy of phasing.

In accordance with the benchmarking using simulated reads, Haplotypes from HaplotypeTools covered 5X more of the genome (674 kb; 2.9%) than GATK physical phasing alone, while WhatsHap covered 7.4X more of the genome (5 Mb; 21.4%) than GATK alone (135 kb; 0.6%). Haplotypes were also longer with both HaplotypeTools (N_max_ 1.1 kb, N_50_ 115nt) and WhatsHap (N_max_ 8.5 kb, N_50_ 1.1 kb) compared with GATK alone (N_max_ 230 nt, N_50_ 26 nt). HaplotypeTools phased genomic regions overlapped with WhatsHap by 98%. Only 7% of HaplotypeTools phased genomic regions were also phased by GATK, primarily reflecting the fewer sites phased by GATK.

**Fig. 2:**
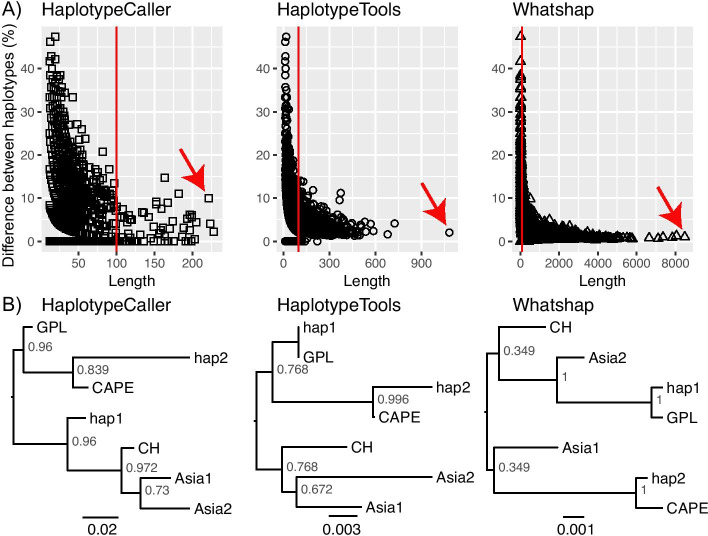
Comparisons of haplotypes for the hybrid *Bd* isolate SA-EC3, generated by GATK v4 HaplotypeCaller physical phasing, HaplotypeTools and WhatsHap. **A** The length in nucleotides of haplotype pairs vs the difference between those haplotypes pairs (%). The red line indicates the minimum haplotype length used for all analysis, and the red arrow indicates the haplotype pairs illustrated in part B of this figure. **B** HaplotypeTools’ utility script HaplotypePlacer constructs haplotype trees with FastTree. Among the longest representative haplotype pairs (shown by the red arrow) are shown including supercontig 1.7 positions 1,335,923–1,336,143 for HaplotypeCaller, supercontig 1.17 positions 206,141–207,223 for HaplotypeTools and supercontig 1.4 positions 319,655–328,139 for WhatsHap. In each of these examples, one haplotype is closest to *Bd*CAPE. For HaplotypeTools and WhatsHap that have several advantages over HaplotypeCaller’s physical phasing presented, the second haplotype is closest to *Bd*GPL

Phased SA-EC3 VCF’s from each tool were compared to consensus genomes for each lineage (generated by a HaplotypeTools utility script). First, pairs of haplotypes from single locations were compared for length and sequence similarity (Fig. 2A) demonstrating that most of the haplotypes in all tools were short and as a proportion of their total length, contained a greater number of nucleotide differences between them. The longest and most divergent haplotypes are the most informative in terms of ancestry, and example haplotypes and their phylogenetic placement are shown for each tool for illustrative purposes only (Fig. 2B). One haplotype is phylogenetically closest to *Bd*CAPE for all three haplotype-based trees chosen. For trees based on HaplotypeTools and WhatsHap haplotypes, the second haplotype is closest to *Bd*GPL, while the second haplotype is *Bd*CH for HaplotypeCaller physical phasing alone.

**Fig. 3:**
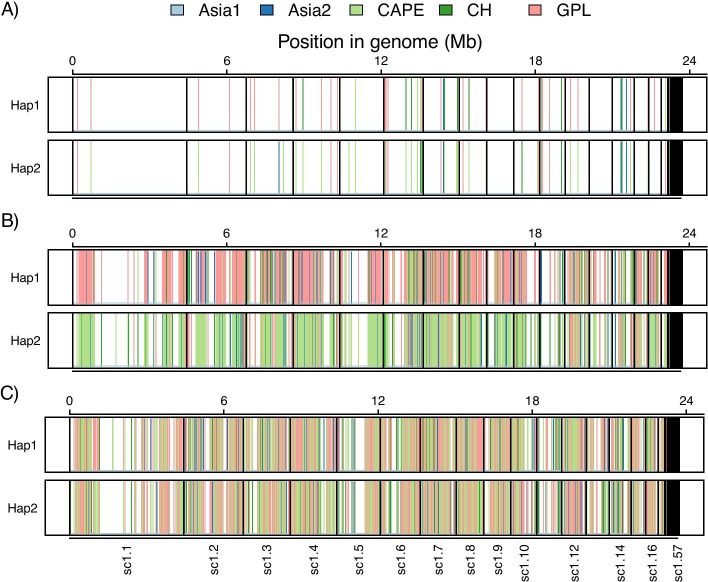
HaplotypeTools’ utility script HaplotypePlacer was used to produce 10 kb windows showing the genomic region for each haplotype pairs and colored according to their closest relative (light blue = *Bd*Asia1, blue = *Bd*Asia2, light green = *Bd*CAPE, green = *Bd*CH, red = *Bd*GPL). Windows are for **A** GATK v4 HaplotypeCaller, **B** HaplotypeTools and **C** WhatsHap

The HaplotypeTools utility script HaplotypePlacer iteratively constructs approximately-maximum-likelihood phylogenetic trees using FastTree for every haplotype (with a default 100 nt minimum haplotype length parameter) in order to identify overall trends in haplotype relatedness to other lineages or species. HaplotypePlacer also outputs a summary for the closest relative across all the haplotypes (Table 4) and generates non-overlapping window plots showing the genomic region for each haplotype pairs, which are colored according to their closest relative (Fig. 3). Using this iterative approach for every haplotype, the majority of haplotypes from hybrid *Bd* isolate SA-EC3 were phylogenetically clustered with *Bd*GPL for one of the haplotypes and *Bd*CAPE for the other, confirmed by each of the three tools tested (65% for HaplotypeCaller, 80% for HaplotypeTools and 91% for WhatsHap). Therefore, the parental lineages of *Bd* SA-EC3 are most likely *Bd*GPL and *Bd*CAPE.

**Table 4:** HaplotypeTools’ utility script HaplotypePlacer constructs haplotype trees with FastTree and identifies the closest relative to each

Lineage	***Bd***Asia1	***Bd***Asia2	***Bd***CAPE	***Bd***CH	***Bd***GPL
HaplotypeCaller (nt)	2556	1637	5180	2418	7089
HaplotypeCaller (%)	14	9	27	13	38
HaplotypeTools (nt)	52,806	55,341	3,22,642	51,476	3,14,161
HaplotypeTools (%)	7	7	41	6	39
Whatshap (nt)	3,86,839	2,58,519	43,96,845	2,61,089	44,56,306
Whatshap (%)	4	3	45	3	46
Overlapping phase groups	889	940	1344	758	1018
Overlapping phased positions (OPP)	1941	2131	3457	1661	2487
OPP Same phase (nt)	1758	1922	3421	1486	2467
OPP Same phase (%)	91	90	99	89	99
OPP Cross-over (nt)	183	209	36	175	20
OPP Cross-over (%)	9	10	1	11	1

Hybrid *Bd* isolate SA-EC3 haplotypes from GATK v4 HaplotypeCaller physical phasing, HaplotypeTools and WhatsHap were analysed using HaplotypePlacer, finding that the majority of haplotypes from each of the three tools are closest in those trees to *Bd*GPL (38–46%) and *Bd*CAPE (27–45%). A HaplotypeTools utility script was used to compare phasing between SA-EC3 and each of the lineages. For each comparison, the script identified overlapping phase groups, comprising overlapping phased positions (OPP), which were either in the same phase, or showed evidence of crossovers

To explore recombination in SA-EC3, phased regions between SA-EC3 and phased representatives for each of the lineages were compared using other HaplotypeTools utility scripts (Table 4). The parental lineages identified by HaplotypePlacer (*Bd*GPL and *Bd*CAPE) had the highest number of overlapping phase groups compared with other lineages (1018–1344 compared to 758–940) and highest number of overlapping phased positions/nucleotides (OPP; 2487–3457 compared to 1661–2131), corroborating those lineages as parental lineages, given a greater sequence divergence result in fewer conserved heterozygous positions that can be phased. Only 20 crossovers were detected between SA-EC3 and *Bd*GPL (0.8% of all OPP), and only 36 crossovers were detected between SA-EC3 and *Bd*CAPE (1% of all OPP), compared with 9.43–10.54% for the other lineages, which again supports those relationships, given a greater divergence time may result in greater numbers of ancestral crossovers.

Crossovers between SA-EC3 and its parental lineages were distributed across the genome. For example, SA-EC3 and *Bd*GPL JEL423 had five OPP’s in one overlapping phase group between supercontig 15 positions 473,179–477,410. These phased positions included the following haplotype variant positions for SA-EC3: C-C-**A**-A-A and T-A-**G**-G-C, and for *Bd*GPL JEL423: C-C-**G**-A-A and T-A-**A**-G-C, indicating a crossover in the middle position (473,194), which is located in an intergenic region between hypothetical protein BDEG_28268 and hypothetical protein BDEG_28269 with PFAM Cytochrome P450. The very low levels of crossovers identified between either parent indicate that the parental haplotypes have remained physically separated, suggesting that SA-EC3 is a result of mitotic recombination/parasexuality i.e. genetic exchange without meiosis, and those few crossovers likely resulting from either (1) double mutations, and (2) mitotic gene conversion events.

## Discussion

Correctly identifying haplotypes is central to understanding diploid organisms, including determining genotype–phenotype associations, identifying recombinant or hybrid isolates in microbial populations, determining parental ancestry, and a precursor to a range of population genetic tests. Here, I present a new toolset called HaplotypeTools that is able to accurately phase heterozygous positions from short or long whole genome sequencing data in a fungal genome, and perform a variety of processing steps to recover FASTA files of haplotypes, plot haplotype relatedness to other species across genomic windows, and identify loci of potential crossovers between isolates. HaplotypeTools achieved greater accuracy than two other tools tested (GATK v4 HaplotypeCaller physical phasing and WhatsHap [14]), while also highlighting room for further improvement including computational speed, haplotype length and benefiting from additional data-types such as Hi-C. Currently, regions lacking alignment data or variant calls due to complex genomic regions (such as very repeat rich regions) are ignored by HaplotypeTools, and present a further challenge and opportunity for development.

HaplotypeTools was tested on the hybrid *Bd* isolate SA-EC3 from the Amahlathi Local Municipality of the Eastern Cape in South Africa [19]. Comparing the output of HaplotypeTools to GATK HaplotypeCaller physical phasing revealed that HaplotypeTools was able to recover ~ 5X the total phased nucleotides, and > 4X the haplotype length in terms of haplotype N_max_ and N_50_. HaplotypeTools phased regions were almost entirely contained within WhatsHap [14] phased regions, and based on simulation data, HaplotypeTools phased regions were likely to include a greater number of true positives and fewer false positive phased sites. Phylogenetic placement of those haplotypes by HaplotypePlacer indicated that SA-EC3 is a recombinant of *Bd*GPL and *Bd*CAPE haplotypes, which was supported by all three tools tested. South Africa has endemic *Bd*GPL and *Bd*CAPE lineages present [20], thereby facilitating such an event. ~ 1% of overlapping phased positions indicated crossovers between SA-EC3 and either parental lineage, indicating that meiotic recombination has not occurred between the parental lineages, and the recombinant genotype is more likely to have arisen via mitotic recombination/parasexuality: a process characterised in disparate fungal relatives [21]. *Bd* recombining via parasexuality is parsimonious with polyploidy isolates commonly found [22], and has been hypothesized previously [23]. These results highlight the threat of emerging novel genotypes of pathogens following anthropomorphic spread [19].

HaplotypeTools is designed for phasing bi-allelic data, with tri-alleles phasing a possible upgrade route in the future. However, as shown in the real data experiments, HaplotypeTools works on polyploid genomes and over aneuploidies. HaplotypeTools also has no clear upper limit on genome size or depth of coverage. HaplotypeTools has been tested using variant calls from GATK v4 HaplotypeCaller [12] and Pilon [18], although additional variant callers such as FreeBayes [24] that output in standard VCF should also work, as should other alignment and sequencing strategies. Sequencing technologies Nanopore and PacBio have not been tested with HaplotypeTools, although simulated long reads have been, which showed a particular advantage in accuracy tests. Indeed, given the shorter haplotypes offered by HaplotypeTools, a particular strength may be high accuracy haplotypes stemming from such long-read data. Sequencing strategies that yield higher sequencing errors could be accommodated by adjusting the ‘cut-off percent reads supporting phase group’ parameter. Lower sequencing depth could be accommodated by adjusting minimum read depth. Where the use case is very different from those presented here, the tools to perform accuracy checks have been included in the HaplotypeTools toolset, and ideally will be used to validate phasing accuracy and thereby optimized for individual use cases.

The interpretation and usefulness of HaplotypePlacer will rely on the lineages or species that the phased isolate is compared to. For example, it is advisable that a comprehensive set of possible parental lineages are included in the analysis or HaplotypePlacer will be unlikely to yield a clear answer. The phylogenetic relationships from HaplotypePlacer are not currently tested for significance, and therefore for more robust results, haplotype trees should be examined individually and further phylogenetic tests and tools applied to the multiple alignments output. Future areas of development may include updates to efficiency and computational speed, as well as exploring where haplotypes could be extended further without impacting accuracy, and expanding the toolset to include new tools for population genetic tests such as Four-gamete tests.

## Conclusions

HaplotypeTools is powerful resource that is able to accurately phase and extract haplotypes for population genetic tests and can determine parental ancestry for hybrid or recombinant diploid isolates or individuals. The toolset will be useful for benchmarking new tools or parameter space for phasing accuracy, and visualizing haplotype coverage across a genome and their phylogenetic placement. Therefore, HaplotypeTools should prove valuable for a range of research questions in model and non-model organism genomes.

## Availability and requirements

Project name: HaplotypeTools.

Project home page: https://github.com/rhysf/HaplotypeTools.

Operating systems: Linux, MacOS.

Programming language: Perl and Python.

Other requirements: Perl modules (Bioperl [25], BIO::DB::HTC, Hash::Merge) and Samtools [26]. HaplotypePlacer and associated scripts currently require installations of R and several modules (plyr, RColorBrewer) and FastTree [27].

License: MIT License.

Any restrictions to use by non-academics: Not applicable.

## Methods

### HaplotypeTools algorithm

The algorithm for HaplotypeTools comprises on five steps. The first step splits the VCF into windows of a specified length (default 10 kb), and BAM files into windows of the same length. Step 3 combines pairs of BAM and VCFs for each sample by assigning read information to intermediate VCF files (i.e. VCF-[contig]-[start window]-[stop window]-phased-[sample number]). Step 4 assigns phase groups based on 5 conditions, outputting intermediate tabulated files (i.e. VCF-[contig]-[start window]-[stop window]-phased-[sample number]-and-assigned.tab). Step 5 merges all phased samples for a given window into VCFs (i.e. VCF-[contig]-[start window]-[stop window]-phased), and then concatenates those into a final phased VCF. Splitting input data into windows allows steps 3 and 4 to be run in parallel on a cluster (Platform Load Sharing Facility (LSF), Sun GridEngine (SGE) or Univa GridEngine (UGE) currently supported). HaplotypeTools can also be run on an individual computer in serial at the expense of slower computational time.

Step 3 of HaplotypeTools assigns Phase Positions (PP) for all reads that overlap ≥ 2 heterozygous positions, which are separated by semicolons and stored in the ID column of the output. PPs consist of:

1. unique read count (RC) and
2. read genotype values (rGT) or read nucleotide values (rNT)

RCs serve as simple integer identifiers (0, 1, 2, *n*) for step 4 to identify reads that overlap multiple VCF positions, the value of which is incremented for each new read in the BAM. rGT are variant positions in sequence reads corresponding to a sequence match to the VCF REF column (0) or ALT column (1,2 etc.). rNT are used instead of rGT for variant positions in sequence reads that do not match a VCF REF or ALT base (based on the CIGAR flag), e.g. rNT = A or rNT = ATCC. For example, following step 3, an ID column could be’0-PP-0;1-PP-0;2-PP-1;3-PP-1;4-NT-A’, indicating 5 reads align over this VCF position in total, two of which have the REF allele, two that have the ALT allele, and one that has an adenosine, which is not described in the VCF REF or ALT column at that position.

Step 4 runs through pairs of consecutively found heterozygous positions named Previous Heterozygous Position (PHP) and Current Heterozygous Position (CHP), checking them for 5 conditions:

1. Check for ≥ 2 rGT’s in CHP.
2. Check the 2 CHP rGT’s with the highest depth > min. haplotype depth parameter.
3. Check the 2 CHP rGT combined depth (percent) > phase cutoff parameter.
4. Check PHP passed conditions 1–3.
5. Check for ≥ 2 haplotypes from PHP and CHP PPs.

If any of those 5 conditions are not fulfilled, the PHP ID column is replaced by a comment stating the sample number and the reason it was not phased. A Phase Block (PB) integer value (identifier for separate haplotypes) is also incremented. The following pair of PHP and CHP are then assessed. Providing all 5 conditions are met, the reads that match the two PHP rGT’s and the two CHP rGT’s are identified, and used to construct a new CHP phased genotype. In the case > 2 rGT’s are found, the two with the highest depth are selected. A phase group (PG) is assigned to PHP (if not already assigned) and CHP, which is appended to the SAMPLEINFO column. The PG consists of the contig, sample number, start window and PB separated by dashed (e.g. supercont1.1-0-350,000-1), ensuring every PG is unique e.g. the same phase block identifier in the same window (350,000–360,000) for a 2nd sample in a multi sample VCF will be supercont1.1-1-350,000-1. A summary file for each window is printed including contig, position, ID and SAMPLEINFO, which is used to update the final phased VCF during concatenation in Step 5.

### Benchmarking using simulated data

HaplotypeTools and WhatsHap (downloaded from https://github.com/whatshap on 1st March 2021) were benchmarked using simulated data from the *Batrachochytrium dendrobatidis* (*Bd*) JEL423 genome. First, 40 contigs each of < 10 kb were removed from the reference sequence, ensuring we could simulate 10 kb reads across the genome (updated reference = 29 contigs, 23.44 Mb, N50 = 1.7 Mb). Next, the Biscap utility script “Introduce Random Mutations (IRMS)” [28] was used with the heterozygous setting (HET), which duplicates every chromosome (homologous versions), followed by selecting random nucleotides to ‘mutate’ into other random nucleotides across both chromosomes and homologous chromosomes. Three such modified reference genomes were generated including 1 SNP/Kb (23,137 total), 10 SNP/Kb (231,370 total) and 100 SNP/Kb (2,313,700 total). Next, sequence reads were simulated from this duplicated and modified reference sequence using WGSim (https://github.com/lh3/wgsim) to ~ 20X depth using either short (100 nt) paired reads (2,313,797 pairs) or long (10 kb) paired reads (23,138 pairs) with no introduced errors (-r 0). Aligning these reads back to the unduplicated and unmodified reference genome will then appear to contain heterozygous positions, for which each position changed is known (the truth set). Reads were aligned to the genome using BWA v0.7.4-r385 mem, and a clean BAM created using Samtools v1.8 view -b -h -f 0 × 2. For WhatsHap compatibility, Picard AddOrReplaceReadGroups was applied to the clean BAM files.

Variants were called from the simulated data alignments using Pilon v1.9 with the diploid flag [18]. For WhatsHap compatibility, reference bases were removed from the VCF. GATK v.4.1.2.0 [12] was not used for calling heterozygous positions from simulated data alignments owing to failing the StrandBiasBySample filter used by HaplotypeCaller. Accuracy of Pilon was assessed using Biscap utility script “Comparison of FDR (CFDR)” [28].

Phased VCF’s from both HaplotypeTools and WhatsHap were assessed for accuracy using HaplotypeTools utility scripts. Specifically, Phased in Any (PIA) regions were identified (VCF_phased_to_PIA.pl), with parameter -t PS for WhatsHap and -t PID (default) for HaplotypeTools. FASTA sequences of haplotypes blocks/pairs were extracted using VCF_phased_and_PIA_to_FASTA.pl. Accuracy was assessed using Haplotype_FASTA_files_to_compare_to_IRMS_het_sites.pl, which calculates for every haplotype block/pair the number of sites that are correctly phased, sites that are incorrectly phased (False Positive type 1) and sites that have been incorrectly variant called and also been phased (False Positive type 2), false negatives within haplotype blocks (not presented), and Switch Errors (incorrect crossovers between haplotypes). To calculate Switch Errors, false negatives were ignored, while False Positive type 2 were considered as a switch error. Additionally, the script produces two summary statistics including overall Switch Error Rate, where the switch error is divided by the number of opportunities for switch errors. Finally, the Quality adjusted N50 (QAN50) was calculated for each test, where each haplotype block/pair is divided into sub-blocks with no switch errors, which are multiplied to the proportion of phased alleles inside that block (called an adjusted span), sorted from largest to smallest, and then the QAN50 is the size of the adjusted span that includes more than half of the total variants [29].

### HaplotypeTools using real data

To test HaplotypeTools on real data, variant calling was first applied to a major fungal pathogen of amphibians, *Batrachochytrium dendrobatidis*, which has a 23 Mb diploid or triploid (with frequent aneuploid [22]) genome. Paired-end Illumina data from representatives of all five known lineages (*Bd*GPL JEL423, *Bd*CAPE TF5a1, *Bd*CH ACON, *Bd*Asia-1 KRBOOR_323, *Bd*Asia-2 CLFT065, and a hybrid of unknown parentage SA-EC3) were obtained from the NCBI Sequence Read Archive (SRA) [19, 22, 30]. The Genome Analysis Toolkit (GATK) v.4.1.2.0 [12] was used to call variants. Our Workflow Description Language (WDL) scripts were executed by Cromwell workflow execution engine v.48 [31]. Briefly, raw sequences were pre-processed by mapping reads to the reference genome *Bd* JEL423 using BWA-MEM v.0.7.17 [32]. Next, duplicates were marked, and the resulting file was sorted by coordinate order. Intervals were created using a custom bash script allowing parallel analysis of large batches of genomics data. Using the scatter–gather approach, HaplotypeCaller was executed in GVCF mode with the diploid ploidy flag. Variants were imported to GATK 4 GenomicsDB and hard filtered (QD < 2.0, FS > 60.0, MQ < 40.0, GQ ≥ 50, AD ≥ 0.8, DP ≥ 10). HaplotypeTools and WhatsHap were used individually to phase each VCF with default parameters, and HaplotypeTools utility scripts to phylogenetically place and visualise haplotype placement across the genome, as well as explore crossovers between pairs of phased VCFs.

## Data Availability

HaplotypeTools is open source and freely available from https://github.com/rhysf/HaplotypeTools. The *Bd* genome is available from NCBI (PRJNA13653). DNAseq for isolates JEL423, KRBOOR_323, CLFT065 and SA-EC3 are available from NCBI (PRJNA413876). DNAseq for isolates TF5a1 and ACON, are available from NCBI (PRJNA174849).
